# The Role of Gasotransmitter-Dependent Signaling Mechanisms in Apoptotic Cell Death in Cardiovascular, Rheumatic, Kidney, and Neurodegenerative Diseases and Mental Disorders

**DOI:** 10.3390/ijms24076014

**Published:** 2023-03-23

**Authors:** Stanislav Rodkin, Chizaram Nwosu, Alexander Sannikov, Anton Tyurin, Vasilii Sergeevich Chulkov, Margarita Raevskaya, Alexey Ermakov, Evgeniya Kirichenko, Mitkhat Gasanov

**Affiliations:** 1 Faculty of Bioengineering and Veterinary Medicine, Department of Bioengineering, Don State Technical University, Rostov-on-Don 344000, Russia; 2 Department of Psychiatry, Rostov State Medical University, Rostov-on-Don 344022, Russia; 3 Internal Medicine Department, Bashkir State Medical University, Ufa 450008, Russia; 4 Department of Faculty Therapy, South-Ural State Medical University, Chelyabinsk 454092, Russia; 5 Department of Internal Diseases #1, Rostov State Medical University, Rostov-on-Don 344022, Russia

**Keywords:** gasotransmitters, apoptosis, nitric oxide, carbon monoxide, hydrogen sulfide, sulfur dioxide, cardiovascular diseases, rheumatic diseases, kidney diseases, neurodegenerative diseases, mental disorders, cytoprotection

## Abstract

Cardiovascular, rheumatic, kidney, and neurodegenerative diseases and mental disorders are a common cause of deterioration in the quality of life up to severe disability and death worldwide. Many pathological conditions, including this group of diseases, are based on increased cell death through apoptosis. It is known that this process is associated with signaling pathways controlled by a group of gaseous signaling molecules called gasotransmitters. They are unique messengers that can control the process of apoptosis at different stages of its implementation. However, their role in the regulation of apoptotic signaling in these pathological conditions is often controversial and not completely clear. This review analyzes the role of nitric oxide (NO), carbon monoxide (CO), hydrogen sulfide (H_2_S), and sulfur dioxide (SO_2_) in apoptotic cell death in cardiovascular, rheumatic, kidney, and neurodegenerative diseases. The signaling processes involved in apoptosis in schizophrenia, bipolar, depressive, and anxiety disorders are also considered. The role of gasotransmitters in apoptosis in these diseases is largely determined by cell specificity and concentration. NO has the greatest dualism; scales are more prone to apoptosis. At the same time, CO, H_2_S, and SO_2_ are more involved in cytoprotective processes.

## 1. Introduction

Cardiovascular, rheumatic, kidney, and neurodegenerative diseases (hereinafter referred to as internal diseases) and mental disorders are the most common diseases worldwide that can lead to disability and death. Their development requires a deep understanding of the molecular and cellular processes underlying these pathological conditions. However, many of the intracellular signaling mechanisms that are realized in internal diseases and mental disorders are still poorly understood. Over the past decades, a lot of scientific data have accumulated, which indicate that gasotransmitters play an important role in the pathogenesis of these diseases, as well as in the process of cell death, which may be associated with them, in apoptosis [1,2,3,4,5].

Apoptosis is the most common form of programmed cell death, characterized by a complex set of biochemical and molecular genetic changes in the cell as a result of which it breaks up into separate apoptotic bodies bounded by the plasma membrane. The induction of apoptosis can be caused by both internal and external physiological and pathological factors. It should be taken into account that apoptosis is an integral part of the normal functioning of the body. However, the redundancy of this process underlies many pathological conditions [6].

Today, it is known that an important role in the regulation of apoptosis is played by gasotransmitters—a new class of gaseous signaling molecules that perform a wide variety of functions in the body, ranging from the regulation of vascular tone to subtle mechanisms of neuromodulation [7]. So far, four gasotransmitters have been discovered: nitric oxide (NO), carbon monoxide (CO), hydrogen sulfide (H_2_S) [8], as well as sulfur dioxide (SO_2_) [6,9]. All of them are a necessary link in maintaining intracellular homeostasis. However, these messengers are also involved in the pathogenesis of internal diseases and mental disorders. However, the role of NO, CO, H_2_S, and SO_2_ in the regulation of apoptotic signaling in these pathological conditions is often controversial and has not been fully studied. 

In our studies, we have already studied gasotransmitters in neurodegenerative processes in the nervous tissue associated with axotomy [10] and photooxidative stress [11]. Having experience in studying this class of molecules, we observed the lack of a unified concept of their role in apoptosis and the inconsistency of scientific results in internal diseases and mental disorders, hence we decided to conduct this study.

Therefore, the purpose of this review was a large-scale analysis of gasotransmitter-dependent processes associated with apoptosis in cardiovascular, rheumatic, kidney, and neurodegenerative diseases and mental disorders. Consideration and comparison of a large amount of literature data on the role of these messengers in apoptosis in these pathological conditions will allow a better understanding of their role in cell survival and death as well as help to choose the right strategy for further research in this area.

## 2. Apoptosis

One of the types of programmed cell death is apoptosis, which is constantly realized in the body; however, under conditions of deviation from the norm, it can act as a negative regulator of the progression of the pathological condition. As a result of apoptosis, the cell breaks up into separate apoptotic bodies bounded by the plasma membrane. Fragments of a dead cell quickly undergo the process of phagocytosis, bypassing the development of an inflammatory reaction [12].

External and internal factors, such as hypoxia, disruption of cell cycle signals, DNA damage, oxidative stress, chemical agents, physical impact, etc., can act as inducers of apoptotic signaling. Despite the variety of factors that induce this process, there are two signaling pathways of apoptosis: a receptor-dependent (external) signaling pathway involving death receptors and a mitochondrial (internal) pathway [13].

### 2.1. Receptor-Dependent Pathway of Apoptosis

**Figure 1:**
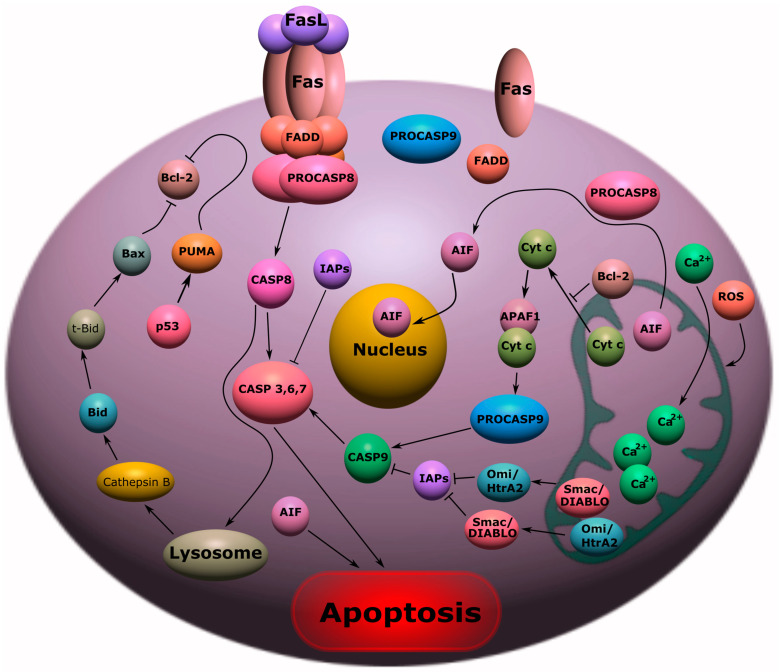
Signaling pathways of apoptosis: extrinsic and intrinsic pathways. Fas, death receptor; FasL, Fas ligand; FADD, Fas-associated death domain; PROCASP8, procaspase 8; PROCASP9, procaspase 9; CASP8, caspase 8; CASP9, caspase 9; CASP 3, 6, 7, caspases 3, 6, 7; Bid, BH3 interacting-domain death agonist; t-Bid, truncated Bid; Bax, bcl-2-like protein 4; Bcl-2, B-cell lymphoma 2; PUMA, p53 up-regulated modulator of apoptosis; p53, tumor suppressor protein; IAPs, inhibitor of apoptosis protein; Omi/HtrA2, mitochondrially-located serine protease; Smac/DIABLO, second mitochondria-derived activator of caspase/direct IAP Binding protein with Lowp I; Cyt c, cytochrome c; APAF1, apoptotic protease activating factor 1; AIF, apoptosis inducing factor; Ca^2+^, calcium ions; ROS, reactive oxygen species. Arrows with a sharp end—positive regulation; arrows with a blunt end—negative regulation.

Initiation of the external pathway of apoptosis occurs by binding of death ligands to membrane Fas or TNF1 death receptors, which leads to their activation and recruitment of adapter proteins FADD (Fas-associated death domain) [14] or TRADD (TNF receptor-associated death domain), respectively. As a result, a death-inducing signaling complex (DISC-death-inducing signaling complex) is formed [15], which initiates the assembly and activation of the initiator caspase-8, which triggers a cascade of effector caspases (caspases-3, -6, -7) (Figure 1) [13,14,15].

Effector caspases cleave the musculoskeletal structures of the cell, inhibit protein biosynthesis, and activate endonuclease [16]. Caspase-8 can stimulate the release of cathepsin from lysosomes, which leads to the activation of Bax, which either inhibits Bcl-2 or forms a complex with porin. This increases the permeability of the outer mitochondrial membrane and promotes the release of the key protein of the mitochondrial apoptosis pathway, cytochrome c (Cyt c) (Figure 1) [17].

### 2.2. Mitochondrial Pathway of Apoptosis

The mitochondrial pathway of apoptosis is based on the regulation of mitochondrial outer membrane permeabilization (MOMP) by proteins of the Bcl-2 family. Proteins Bcl-2, Bcl-W, Bcl-XL, MCL-1, and Bfl-1 suppress apoptosis by blocking the mitochondrial release of cytochrome c. Apoptosis is stimulated by the proapoptotic proteins Bik, Bcl-Xs, Bad, Bax, Bak, Bid, Bim, and Hrk, which increase MOMP. Mitochondrial Ca^2+^ overload and development of oxidative stress underlie this process. As a result, Cyt c enters the cytoplasm, which activates caspase-9 through the formation of an apoptosomal complex with Apaf-1 (apoptotic protease activating factor 1) (Figure 1) [13,18].

Apoptosis can be modulated by a number of proteins released from mitochondria into the cytoplasm: Smac (second mitochondria-derived activator of caspase)/DIABLO (direct IAP Binding protein with Lowp I) [19], HtrA2/Omi [20], and others. They bind apoptosis suppressors, proteins of the IAP family (inhibitor of apoptosis protein), which, in turn, are capable of inhibiting caspases-3, -7, and -9 [21]. AIF (apoptosis inducing factor) can induce caspase-independent apoptosis, causing chromatin condensation and DNA cleavage [22]. One of the key proteins of the mitochondrial pathway of apoptosis is the p53 protein, which can lead to the expression of many proapoptotic proteins, for example, PUMA (Figure 1) [23].

## 3. Gasotransmitters and Their Role in Apoptosis

Gasotransmitters are a new class of gaseous messengers that perform signaling functions in the cell and participate with high specificity in intercellular and intracellular processes. This class of molecules is formed in the body endogenously under the action of the corresponding enzymes. Unlike canonical transmitters, they are small molecules, which allows them to easily diffuse through biological membranes, and their high reactivity allows them to interact with a wide range of molecular targets. The classic triad of gasotransmitters includes nitric oxide (NO), carbon monoxide (CO), and hydrogen sulfide (H_2_S) [8]. However, relatively recently, another gas mediator was discovered—sulfur dioxide (SO_2_) [6,9].

These universal secondary messengers are involved in normal and pathological conditions, tipping the balance either towards cell survival or cell death [6,8,9].

It is known that gasotransmitters can regulate apoptotic signaling. Their role in this process is especially pronounced in conditions of pathological disorders in the body. Depending on the type of gasotransmitter, various signaling mechanisms can be realized that activate or inhibit apoptosis. The pathological process itself, in which one or another gaseous second messenger is involved, is also important [7].

### 3.1. Nitric Oxide

**Figure 2:**
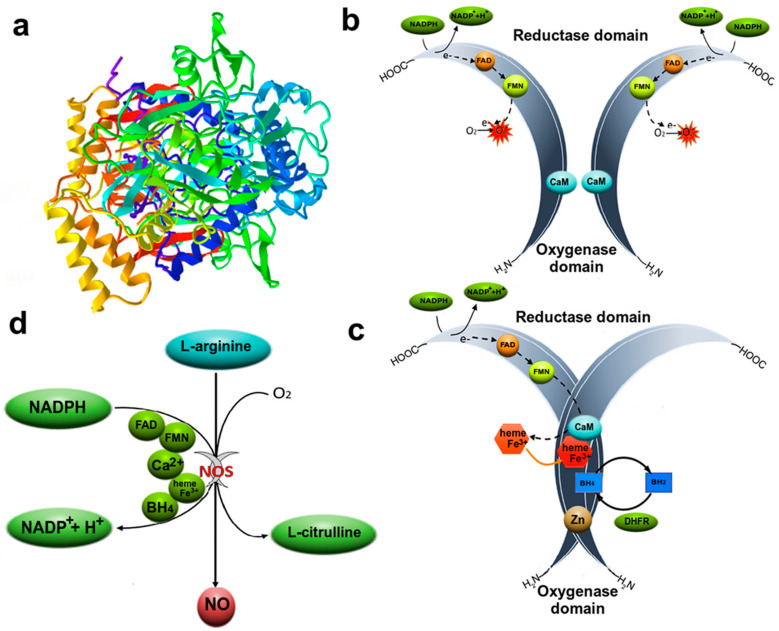
NO synthase structure and biocatalysis. (**a**) 3D-model of the structural organization of nNOS. (**b**) Monomeric organization of NOS: In NOS monomers, electron transfer can occur from reduced NADPH to FAD and FMH, resulting in the reduction of molecular oxygen to superoxide (O_2_^−^). Monomers and isolated reductase domains can bind to calmodulin (CaM). NOS monomers cannot bind to the cofactor tetrahydrobiopterin (BH_4_) or the substrate L-arginine, making NO generation impossible. (**c**) A functional dimer can form in the presence of heme. Heme is required for cross-domain electron transfer from flavins to heme of the opposite monomer. (**d**) NO biosynthesis by constitutive Ca^2+^-dependent NOS.

NO is a universal messenger responsible for the relaxation of vascular smooth muscles, neutralization of pathogenic agents, neurotransmission, antitumor activity, etc. Numerous cell types produce NO, from endotheliocytes to neurons and glial cells. NO biosynthesis occurs under the action of NO-synthase enzymes (Figure 2). To date, three main types of NOSs are known: endothelial and neuronal NOSs (eNOS/NOS3, nNOS/NOS1), related to constitutive (calcium-dependent), and inducible NOS (iNOS/NOS2, calcium-independent), activated by cytokines and lipopolysaccharides within a few hours [7,8,24]. NOS isoforms are expression products of different genes and perform different functions. It is believed that at low concentrations, NO has cytoprotective properties, and at high concentrations, in particular, due to the work of iNOS, it causes cell death [24].

NO is able to bind into stable compounds, be deposited in cells, and be transported over long distances in the body. S-nitrosothiols (RSNO) and iron dinitrosyl complexes (DNICs) can act as NO depots. Moreover, DNIC and RSNO can influence many physiological processes [25]. It is known that NO is involved in many pathological processes, such as stroke, myocardial infarction, kidney and liver failure, cancer, Parkinson’s, Huntington’s, Alzheimer’s, multiple sclerosis, schizophrenia, etc. [24]. All these pathological conditions are to some extent associated with cell death, in particular, apoptosis.

Most often, the role of NO in the induction of apoptosis is considered in the context of the intensification of free radical processes, which are in a state of dynamic equilibrium of the oxidant/antioxidant system and are an important and necessary link in metabolism. However, a violation of the coordination of the action of the components of the antioxidant system leads to the development of nitrosyl stress, which underlies many pathological conditions. NO can interact with molecular oxygen, superoxide anion, and transition metals to form reactive nitrogen species, the most aggressive of which is peroxynitrite (ONOO^−^). This radical nitrates amino acids, such as tyrosine, and also oxidizes various biomolecules, including proteins, lipids, and nucleotides [26]. These processes lead to the intensification of lipid peroxidation, depletion of the pool of adenosine triphosphoric acid, impaired function of calcium channels, destruction of the cytoskeleton, mitochondrial dysfunction, etc., which can ultimately trigger apoptosis [27]. For example, hyperproduction of NO can cause Ca^2+^ excitotoxicity through the activation of NMDA receptors and, as a result, lead to Ca^2+^ overload of the cell, an increase in MOMP, and the release of cytochrome c and other proapoptotic factors from mitochondria into the cytoplasm [28].

Along with the crude mechanisms of NO-dependent apoptosis induced by nitrosyl stress, there are subtle processes of regulation of the apoptotic signal by this messenger. NO can directly interact with key proapoptotic proteins, modulating their activity, localization, and expression. Thus, in our recent work, we were able to show that NO can induce in axotomized neurons and surrounding glial cells the nuclear deposition of the p53 transcription factor known as the “guardian of the genome”, which is involved in many cellular functions, including apoptosis [10]. This biological effect may be associated with Tyr327 nitration in the tetramerization domain of p53, which leads to its accumulation in the nuclear region [29]. NO can bind to the phosphorylation sites of the p53 molecule and lead to disruption of its binding to Mdm2, a ubiquitin ligase that marks p53 for degradation by the proteasome [30]. Moreover, NO can modulate the activity and level of E2F1, a transcription factor for many genes, including TP53, the p53 protein gene. NO increases the level of E2F1 through its hyperphosphorylation and pRb inactivation and also increases the DNA-binding capacity of E2F1 through p38 MAPK activation [31]. In addition, caspase-3, which plays a central role in the caspase cascade, can act as a molecular target for NO. It has been shown that NO can inhibit caspase-3 activation through cGMP-dependent and independent mechanisms [32]. NO-dependent S-glutathionylation is another mechanism for regulating the activity of caspase-3, as well as other caspases [33]. S-nitrosylation of the active site of caspase-3 is another NO-dependent regulator of the activity of this enzyme [34]. NO can trigger the mechanisms of apoptotic cell death through activation of p38 MAPK and endoplasmic reticulum stress [35] as well as regulate the expression of Bax, DIABLO, Puma, Apaf-1, and a number of other proteins involved in apoptosis [36]. NO can bind to iron or other metalloproteins that contain transition metals in the active site, modulating their activity and exerting various effects on signaling pathways associated with apoptotic signaling [7,8].

However, the role of NO in apoptosis is far from unambiguous. It, like a “two-faced Janus”, can induce this process or block it through various signaling mechanisms [37].

### 3.2. Carbon Monoxide

CO is a gasotransmitter with a number of biological effects: vasodilation, neurotransmission, inhibition of platelet aggregation, modulation of inflammation, proliferation, cell death, etc. [8,38]. The enzyme responsible for CO synthesis is heme oxygenase (HO), which catalyzes the oxidation of hemoglobin heme [39].

**Figure 3:**
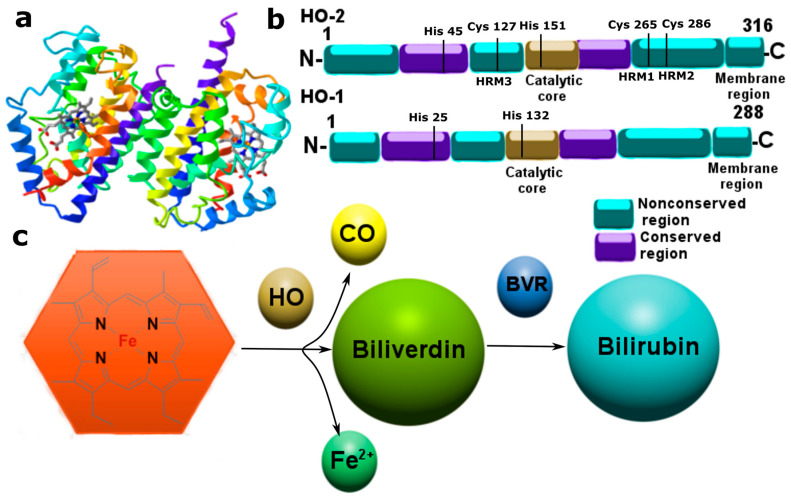
Structural organization of the enzymes responsible for the formation of CO and CO biosynthesis. (**a**) 3D-model of the structural organization of the HO-2 dimer; (**b**) primary structure of HO-1 and HO-2: C-terminal domain required for binding to the cytoplasmic membrane, catalytic domain, and N-terminal cytoplasmic domain. HO-2, unlike HO-1, carries an additional 30 amino acid residues at the N-terminus, as well as two Cys-Pro dipeptides near the C-terminus heme regulatory motifs (HRM); (**c**) schematic pathway of CO biosynthesis: HO catalyzes heme oxidation to form biliverdin CO and Fe^2+^. Biliverdin is converted to bilirubin by the enzyme Biliverdin reductase (BVR).

To date, two isoforms of HO are known, which are present in the cell in the form of an inducible (HO-1) or constitutive (HO-2) form. HO-1 and HO-2 cleave the heme ring in a reaction involving oxygen and NADP with the formation of biliverdin, iron, and CO (Figure 3) [40]. The main source of endogenous CO is the hemoglobin of old erythrocytes [8,40]. The concentration of HO-1 in tissues is at a fairly low level. HO-1 is induced by various cell responses to stress and plays a mainly protective role in damaged tissues, whereas HO-2 is constantly expressed and functions within the normal physiological state. These HO isoforms have an anti-inflammatory effect due to the products of their biocatalysis—bilirubin, which absorbs ROS and inhibits NADP H-oxidase, as well as CO, which relaxes vascular smooth muscles and reduces cell death signals [39]. The HO/CO system is one of the key components of protection against cellular stress caused by ROS, heavy metals, lipopolysaccharides, and other inflammatory factors [41].

It has been established that CO is involved in apoptotic signaling; however, like NO, it has a dual role in this complex process. A number of studies have shown that CO can stimulate or inhibit apoptosis of smooth muscle [42], endothelial [43] and epithelial cells [44], fibroblasts [45], hepatocytes [46], neurons [47], and so on. The CO-dependent mechanisms of the described effects are still poorly understood. However, it is known that endogenous CO induces compensatory expression of antioxidant enzymes through the activation of transcription factors and stress-activated kinases, and also implements other defense mechanisms under conditions of oxidative stress [41,48]. However, under conditions of excessive production of CO, it can itself induce oxidative stress and lead to a serious disruption of intracellular homeostasis, triggering apoptosis. The anti-apoptotic effect of CO may be due to the modulation of Bcl-2. It has been shown that CO induces the expression of this anti-apoptotic protein in neurons [49]. Many effects of CO are due to the high affinity for the reduction of transition metals, for example, Fe^2+^, and the combination of CO with metalloproteins is quite stable and causes various molecular cellular events, including those leading to cell death [41]. CO-induced apoptosis may be due to the activation of the FADD protein and caspase-8, -9, and -3 [50]. It is known that CO at a high level can lead to blocking of electron transport in mitochondria and energy collapse of the cell [51].

CO signaling is molecular cooperation in a feedback ring with NO, which can induce CO synthesis by regulating mRNA and the HO-1 protein itself. In turn, CO can control the formation of NO. Moreover, it has been shown that CO and NO can simultaneously interact with heme [41]. Undoubtedly, CO is a unique signaling molecule that acts in cooperation with its older brother NO, triggering entire cascades of molecular–cellular events under normal and pathological conditions.

### 3.3. Hydrogen Sulfide

**Figure 4:**
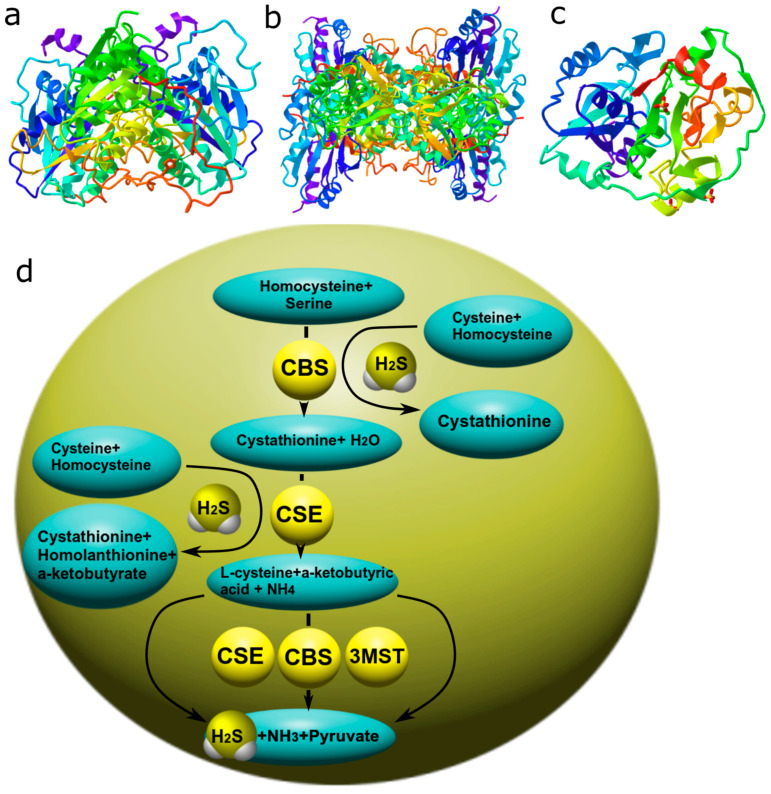
Structural organization of the enzymes responsible for the formation of H_2_S and H_2_S biosynthesis. 3D-model of the structural organization: (**a**) cystathionine-β-synthase (CBS), (**b**) cystathionine γ-lyase (CSE), and (**c**) 3-mercaptopyruvate sulfurtransferase (3-MST). (**d**) Variety of pathways for H_2_S biosynthesis under the action of enzymes CBS, CSE, and 3-MST.

The third gasotransmitter is H_2_S, which is formed in the nervous tissue of the brain [52] and exists in the cell in the form of gaseous molecules or sodium bisulfide. H_2_S is deposited in the form of sulfhemoglobin, as well as in the complex of some proteins [53]. The main substrate for the production of H_2_S in the cell is L-cysteine and cystine. The enzymes responsible for the synthesis of hydrogen sulfide are cystathionine-β-synthase (CBS), cystathionine γ-lyase (CSE), and 3-mercaptopyruvate sulfurtransferase (3-MST) together with cysteine aminotransferase (CAT) [52,53]. The main enzyme for H_2_S synthesis is CBS, which is predominantly expressed in the brain [54]. In turn, CSE also makes a significant contribution to the formation of H_2_S (Figure 4) [55].

H_2_S catabolism can occur by oxidation, methylation, and exhalation [53].

To date, it is known that the functions of H_2_S are not limited to neuromodulation, but much wider, ranging from angiogenesis to complex apoptotic signaling. This messenger is involved in both normal processes and pathological processes associated with stroke, neurodegenerative diseases, diseases of the kidneys, lungs, etc. [53,56,57]. In this regard, the role of H_2_S in apoptosis is of particular interest, and although the scales tilt towards the inhibitory effect of H_2_S on this process, there are studies showing the reverse side of H_2_S—cytotoxicity.

It is known that H_2_S protects the cell from oxidative stress by increasing the level of reduced glutathione (GSH), which is a key antioxidant. It has been shown that H_2_S enhances the transport of cysteine and, as a consequence, the synthesis of GSH, which is a tripeptide consisting of cysteine, glutamate, and glycine. At the same time, H_2_S itself can react and quench the superoxide anion (O_2_^−^), nitric oxide (NO), and its free radical products [58]. In addition, H_2_S increases the level of thioredoxin (Trx-1), an oxidoreductase that exhibits antioxidant properties [59]. H_2_S can also activate a number of other antioxidant defense enzymes, protecting cells from ROS-induced apoptosis [56]. However, a high level of H_2_S can, on the contrary, induce the formation of ROS and cause an increase in oxidative stress.

H_2_S-dependent regulation of apoptosis can be carried out through the modulation of the activity of NMDA receptors, which provide the incoming current of calcium and depolarization of cell membranes. Violation of their functions leads to Ca^2+^ excitotoxicity and cell death of neurons through apoptosis. H_2_S can directly interact with cysteine residues of NMDA receptor subunits through their S-sulfhydration and modulate their activity through a number of signaling pathways, for example, by activating PKA [60]. It should be noted that H_2_S, as in the case of NO and CO, can regulate the level of anti- and pro-apoptotic groups of proteins. Moreover, it can interact with them directly through S-sulfhydration or persulfhydration of cysteine residues and through activation or inhibition of signaling mechanisms. It has been shown that H_2_S can either activate or inhibit the expression of p53 [61,62], caspase-3, Bax, and a number of other proteins involved in apoptosis [56]. However, the subtle H_2_S-dependent mechanisms of regulation of anti- and pro-apoptotic proteins remain poorly understood.

### 3.4. Sulfur Dioxide

Sulfur dioxide (SO_2_) is a recently discovered gasotransmitter that exhibits a number of biological effects: antioxidant, anti-inflammatory, antihypertensive, antiatherogenic, and so on. It was shown for the first time that SO_2_ is endogenously formed in the cardiovascular system and has a pronounced vasorelaxant effect [8]. Later, its products were found in the stomach, liver, lungs, spleen, brain, etc. [63,64]. Aspartate aminotransferase (AcAT) is responsible for the synthesis of SO_2_. SO_2_ catabolism is carried out by hydrogenation of its bisulfite (HSO_3_^−^) and sulfite (SO_3_^2−^) ions, which are further oxidized to sulfate [64,65].

To date, it has been established that SO_2_ is involved in apoptosis and oxidative stress. SO_2_ increases the expression of antioxidant enzymes such as SOD2 and GSH-Px1 and reduces the production of ROS [66]. At the same time, the antiapoptotic effect of SO_2_ may be due to increased expression of Bcl-2 and inhibition of Bax, stabilization of mitochondrial membranes, a decrease in the release of cytochrome c, and a decrease in caspase activation [67]. In addition, SO_2_ is involved in intracellular calcium homeostasis, the violation of which lies in cell death in many pathological conditions [64,65].

## 4. Molecular Mechanisms of Gasotransmitter-Dependent Apoptosis in Internal Diseases

### 4.1. Cardiovascular Diseases

**Figure 5:**
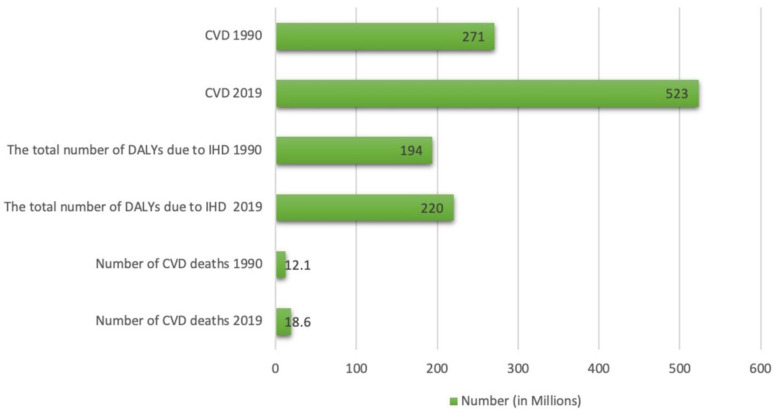
The incidence and mortality of cardiovascular diseases in the world from 1990 to 2019.

Cardiovascular disease (CVD) is the leading cause of death worldwide, despite tremendous progress being made in the medical and surgical treatment of these diseases (Figure 5) [68].

It is known that NO plays an important role in cardioprotection in CVD. It exhibits various biological effects: relaxation of blood vessels, prevention of platelet aggregation, inhibition of leukocyte adhesion, and control of proliferation of vascular smooth muscle cells [69]. NO is produced by resident cardiac cells under stress and in large quantities by activated immune cells that enter the damaged myocardium. A deficiency of eNOS and nNOS exacerbates cardiac injury caused by ischemia/reperfusion or myocardial infarction [70]. NO activates soluble guanylate cyclase (sGC), which leads to an increase in cGMP levels and activation of cGMP-dependent protein kinase (PKG) [71]. In addition, NO induces the opening of mitochondrial K^+^-ATP channels and inhibits Ca^2+^ overload (Figure 5) [72]. In addition, a mechanism has recently been described in which NO protects endothelial cells from oxidative stress-induced apoptosis by inhibiting cysteine-dependent superoxide dismutase (SOD1) monomerization and thus blocking its inactivation [73].

**Figure 6:**
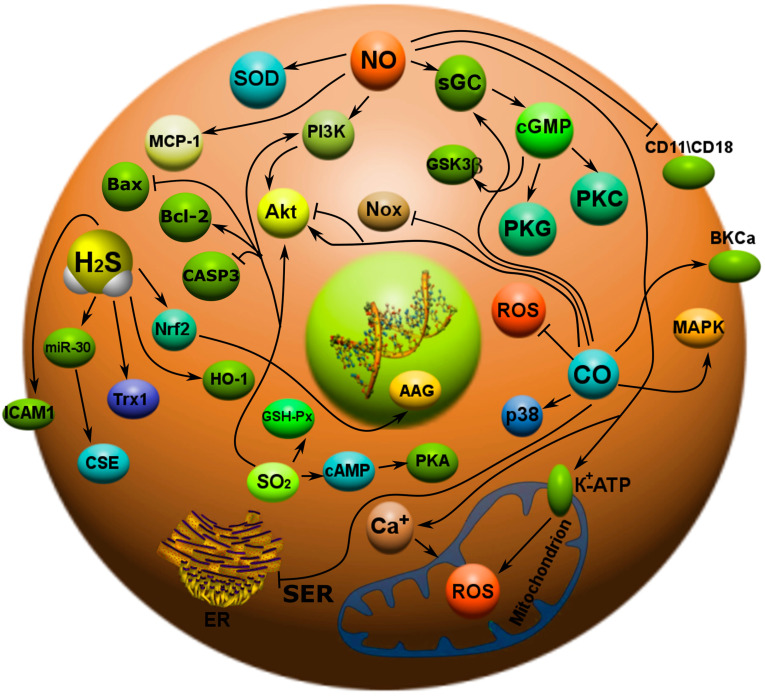
Possible gasotransmitter-dependent signaling mechanisms regulating apoptosis in cardiovascular diseases. NO, nitric oxide; CO, carbon monoxide; H_2_S, hydrogen sulfide; SO_2_, sulfur dioxide; SOD, superoxide dismutase; sGC, soluble guanylyl cyclase; cGMP, cyclic guanosine monophosphate; PKA, protein kinase G; PKC, protein kinase C; PKG, protein kinase G; PI3K, phosphoinositide 3-kinases; Akt, protein kinase B; Bax, bcl-2-like protein 4; Bcl-2, B-cell lymphoma 2; CASP3, cysteine-aspartic acid protease 3; MCP-1, monocyte chemoattractant protein 1; miR-30, microRNA-30; Trx1, thioredoxin 1; CSE, cystathionine γ-lyase; ICAM1, intercellular adhesion molecule 1; HO-1, heme oxygenase 1; GSH-Px, glutathione peroxidase; cAMP, cyclic adenosine monophosphate; MAPK, mitogen-activated protein kinase; p38, mitogen-activated protein kinase p38; BKCa, large conductance calcium-activated potassium channels; CD11\CD18, beta2 integrins, members of the integrin family; Nox, NADPH oxidase; K^+^-ATP, ATP-sensitive potassium channels; ROS, reactive oxygen species; Nfr2, nuclear factor erythroid 2-related factor 2; ER, endoplasmic reticulum; SER, stress endoplasmic reticulum; AAG, anti-apoptotic genes. Arrows with a sharp end—positive regulation; arrows with a blunt end—negative regulation.

The diverse effects of CO are mainly explained by its regulation of general signaling pathways such as stimulation of sGC, opening of Ca^2+^-activated large conductive K^+^ channels (BKCa), and activation of mitogen-activated protein kinase (MAPK) and protein kinase B (Akt). The apoptotic effects of CO are tissue-specific and cell-specific. For example, CO acts as an anti-apoptotic agent in endothelial cells [74] and cardiomyocytes [75], thus preventing cell damage. The anti-apoptotic effects of CO appear to be dependent on p38 activation [43,76], phosphorylation of the protein kinase R-like kinase of the endoplasmic reticulum, and/or via Akt activation [77]. CO has been shown to prevent TNF-α [78] and endoplasmic reticulum (ER) stress-induced apoptosis through a p38 MAPK-dependent mechanism (Figure 6) [74].

H_2_S may be involved in the occurrence and development of some CVDs through various mechanisms [79]. H_2_S has been shown to have a wide range of physiological effects on the cardiovascular system, such as the modulation of blood pressure; effects on angiogenesis, inflammation, and smooth muscle cell growth; apoptosis; antioxidant effects; and cardioprotection [80].

H_2_S can activate Nrf2 signaling to suppress oxidative stress, thereby suppressing atherosclerosis [58]. Nrf2 is known to be an important antioxidant stress transcription factor that regulates the expression of many antioxidant and cytoprotective genes. ROS are an important risk factor for CVD and can induce endothelial cell apoptosis by activating NF-κB, increasing the expression of adhesion molecules and cytokines and enhancing monocytic adhesion (Figure 6) [81,82].

Exogenous H_2_S inhibits endothelial cell autophagy induced by oxidative stress via the Nrf2-ROS-AMPK signaling pathway [83]. The use of H_2_S donors can activate Nrf2 signaling in mice with myocardial ischemia and upregulate the antioxidant HO-1 and Trx1, as well as reduce myocardial ischemic injury. It is assumed that exogenous H_2_S induces nuclear translocation of Nrf2 in cardiomyocytes during myocardial infarction and increases the expression of Trx1 and HO-1 [84].

Great importance is attached to the epigenetic regulation of H_2_S of the cardiovascular system through various mechanisms. Thus, DNA methylation of CSE promoter regions contributes to the development of atherosclerosis or inflammation by reducing CSE transcription and H_2_S production in macrophages [85]. Recent studies show that H_2_S can regulate miRNA expression in CVD. Inhibition of miR-30 can enhance CSE expression and H_2_S production in myocardial ischemia/reperfusion (I/R) rats and counteract myocardial ischemic injury [86]. In neonatal rat cardiomyocytes, NaHS administration can upregulate miR-133a and inhibit cardiomyocyte hypertrophy [87]. Na_2_S administration can increase miR-133a levels and inhibit cardiac muscle cell hypertrophy induced by hyperhomocysteinemia [88]. Overexpression of miR-133a protects against I/R-induced endoplasmic reticulum stress and cardiomyocyte apoptosis [89]. It was also shown that miRNAs can regulate CSE expression in pathological conditions. In the human macrophage THP-1 model, miR-186 directly inhibits CSE expression, which increases macrophage lipid accumulation [90], whereas miR-216a can suppress the expression of CSE and ATP-binding cassette transporter A1 (ABCA1), reducing cholesterol efflux from foam cells [91]. miR-21 overexpression in aortic smooth muscle cells inhibits CSE and specific protein 1 (SP-1) expression, inhibits H_2_S production, stimulates smooth muscle cell proliferation, assembles genes associated with smooth muscle cell differentiation, and regulates CSE/H_2_S-dependent proliferation and differentiation of smooth muscle cells by influencing SP-1 [92]. In a mouse model of myocardial ischemia and inflammation, Na_2_S inhibits myocardial cell apoptosis and necrosis by inducing miR-21 expression, inhibits myocardial inflammation, and reduces infarct size after reperfusion myocardial ischemia [93]. miR-1 attenuates the protective effect of H_2_S on cardiomyocytes by reducing the expression of Bcl-2 [94]. H_2_S increases levels of hypoxia-inducible factor 1-α (HIF1A) via the VEGFR2-mTOR pathway, leading to a decrease in miR-640 levels (Figure 6).

SO_2_ acts as an important regulator of many biological processes in normal and pathological conditions associated with CVD. Recently, studies of the effect of SO_2_ on cell apoptosis have attracted much attention. SO_2_ can regulate the apoptosis of vascular smooth muscle cells, endothelial cells, cardiomyocytes, and a number of other cells that may be involved in the pathogenesis of arterial hypertension (AH) and myocardial damage [95].

ROS play a special role in the regulation of eNOS, which can contribute to the activation of the pro-inflammatory NF-κB-dependent pathway. Under these conditions, NF-κB activation increases the levels of IL-6 and TNF-α cytokines, which can influence tyrosine kinase phosphorylation and decrease NO levels (Figure 6) [96]. Hypertension is accompanied by structural changes in blood vessels, such as hypertrophy and hyperplasia of the walls of blood vessels, which contributes to an increase in vascular resistance. Some NO donors, such as LA-419, have a beneficial effect in preventing the progression of maladaptive cardiac hypertrophy [97].

HO-1 is activated by hemodynamic stress in response to elevated blood pressure. At the same time, the level of HO-1, sGC, and cGMP in vascular smooth muscle cells depends on the stage of development of AH [98]. A number of researchers have demonstrated that CO significantly reduced ventricular hypertrophy and aortic hypertrophy, attenuating the development of angiotensin-dependent type II hypertension in mice. These cardioprotective mechanisms of CO were due to a decrease in ROS production due to a decrease in Nox and Akt phosphorylation [99,100].

Numerous studies show that a decrease in the level of H_2_S contributes to the onset of hypertension. It has been demonstrated in an experimental model that long-term treatment with NaHS can reduce myocardial thickening, coronary intima thickening, interstitial fibrosis, and ROS levels in spontaneously hypertensive rats [101]. Other authors have shown that CSE−/− mice exhibit a significant reduction in endothelium-dependent vasodilation and AH. At the same time, the level of H_2_S in the blood serum, heart, aorta, and other tissues was significantly reduced [102]. Similar results were obtained in children with essential hypertension. Compared with healthy children with normal blood pressure, plasma H_2_S levels in children with essential hypertension were significantly reduced, and systolic blood pressure correlated negatively with the plasma H_2_S/Hcy ratio [103].

SO_2_ can enhance arterial vasorelaxation in spontaneously hypertensive rats by enhancing the vasodilatory response to NO in isolated aortic rings and promoting NO production by aortic cells [104]. Abnormal proliferation of vascular smooth muscle cells induces vascular remodeling and accelerates the development of hypertension. Additionally, SO_2_ significantly inhibits serum-stimulated proliferation of vascular smooth muscle cells by preventing the transition of the cell cycle from G1 to S phase and DNA replication. In addition, SO_2_ increased cAMP synthesis, which led to PKA activation, c-Raf blocking, and extracellularly regulated protein kinase (Erk)/MAPK signaling (Figure 6). As a result, the proliferation of vascular smooth muscle cells was significantly reduced [105].

NO plays an important role in the pathogenesis of atherosclerosis. NO in the endothelium controls the expression of genes involved in atherogenesis. NO reduces the expression of the chemoattractant protein MCP-1 [106]. NO can also inhibit leukocyte adhesion to the vessel wall by reducing leukocyte adhesion molecules CD11/CD18 to bind to the surface of endothelial cells and downregulating CD11/CD18 expression (Figure 6) [107]. Leukocyte adhesion is an early event in the development of atherosclerosis. Endothelial-derived NO prevents endothelial cell apoptosis induced by pro-inflammatory cytokines and pro-atherosclerotic factors, including ROC and angiotensin II. Inhibition of apoptosis may also contribute to the anti-inflammatory and anti-atherosclerotic effect of NO [108]. In addition, NO has been shown to inhibit DNA synthesis, mitogenesis, and proliferation of vascular smooth muscle cells [109]. These antiproliferative effects are likely mediated by cGMP [110]. Inhibition of platelet aggregation and adhesion protects smooth muscle from the effects of platelet-derived growth factors. NO also prevents a later stage of atherogenesis, the formation of fibrous plaque. Based on the combination of these effects, NO produced in endothelial cells can be considered as an anti-atherosclerotic factor [111].

H_2_S has a protective effect on the formation of atherosclerosis. In a knockout mouse model of atherosclerosis apolipoprotein-E (ApoE), plasma H_2_S levels were significantly reduced. Inhibition of CSE further reduced the level of H_2_S and increased the level of intercellular adhesion molecule and plasma-1 (ICAM-1), leading to the progression of aortic lesions. The use of NaHS increased the concentration of H_2_S in plasma, reduced the levels of ICAM-1 in the aorta and plasma, and reduced the area of aortic lesions (Figure 6) [112].

The role of SO_2_ in the development of atherosclerosis was unclear until recently. Plasma and aortic SO_2_ concentrations were reduced in combination with a decrease in aortic aspartate aminotransferase (AAT) activity in atherosclerotic rats [113], indicating a key role for SO_2/_AAT in the pathogenesis of atherosclerosis. The use of SO_2_ donors reduced the size of atherosclerotic plaques in the coronary artery by increasing the level of H_2_S, NO, glutathione peroxidase (GSH-Px), and superoxide dismutase (SOD) in plasma and decreasing the level of malondialdehyde (MDA). Suppression of the proliferation of vascular smooth muscle cells via the cAMP/PKA-mediated Erk/MAPK signaling pathway contributed to the anti-atherosclerotic effects of SO_2_ (Figure 6) [105].

Cardiovascular dysfunction leads to a decrease in NO production in the vessels. During myocardial ischemia reperfusion, more severe cardiac dysfunctions have been found in eNOS-deficient mice compared to wild-type mice [70]. NO is an important modulator of left ventricular remodeling after myocardial infarction. Overexpression of eNOS limits left ventricular dysfunction and remodeling after myocardial ischemia [114]. NO stimulated PKG activity and the opening of K^+^-ATP channels to induce ROS generation in cardiomyocytes [115]. NO prevents the progression of hypertrophy and the development of heart failure through cGMP/GS3Kβ signaling [116]. In the coronary arteries of rats with heart failure, the level of NO was reduced. However, in MI rats, NO levels were increased due to activation of the eNOS/nNOS/PI3K/Akt pathway and decreased ROS formation [117].

Numerous studies have shown that H_2_S can counteract reperfusion myocardial ischemia. In a CSE−/− mouse model, it was shown that H_2_S restores eNOS activity and NO levels in the myocardium, which contributes to the prevention of reperfusion myocardial ischemia [118]. Preliminary use of H_2_S donors can significantly counteract ischemic myocardial injury, reduce the area of myocardial infarction, and reduce troponin-I levels and oxidative stress. It has been shown that H_2_S can increase Nrf2 nuclear translocation and upregulate PKC and STAT-3 phosphorylation by upregulating the expression of HO-1, thioredoxin 1, and heat shock protein 90 (Hsp90) and reducing the activity of proapoptotic factors [84]. NaHS can also reduce caspase-9 activity in cardiomyocytes, increase Bcl-2 expression, reduce p38 MAPK and JNK phosphorylation, and reduce nuclear translocation of p65 NF-κB subunits, which counteracts myocardial reperfusion ischemia [119].

In rat models, it was shown that under conditions of reperfusion MI, SO_2_ preconditioning increased cardiac function and attenuated myocardial cell apoptosis [120]. Ischemic preconditioning-induced endoplasmic reticulum stress (ERS) plays a protective role in ischemic injury. Glucose-regulated protein 78 (GRP78), C/EBP homologous protein (CHOP), and phosphorylation of factor 2 α-subunit (p-eIF2 α) are markers of myocardial ischemia/reperfusion. In addition, SO_2_ preconditioning significantly increased Akt and phosphoinositide 3-kinase (PI3K) p85 phosphorylation and attenuated myocardial injury in rats [95]. Simultaneous enhancement of PI3K/AKT signaling, downregulation of the ERK-MAPK pathway, increase in ERS, enhancement of antioxidant capacity, and attenuation of cardiomyocyte apoptosis may be involved in SO_2_ mediated cardiac defense mechanisms. Apoptosis of cardiomyocytes is a key pathological change in myocardial injury. It should be noted that the use of SO_2_ donors alleviated isoproterenol (ISO^−^)-induced myocardial injury in part by reducing cardiomyocyte apoptosis [67]. The anti-apoptotic function of SO_2_ was mediated by stimulation of Bcl-2 expression, downregulation of Bax expression, increased mitochondrial membrane potential, inhibition of mitochondrial MPTP opening, decreased release of cytochrome C from mitochondria into the cytoplasm, and decreased activation of caspase-9 and caspase-3. SO_2_ can modulate Ca^2+^ current from L-type channels and voltage-dependent K^+^ channels in rat cardiomyocytes. This indicates that ion channels may also be involved in the action of SO_2_ when cardiomyocytes are damaged (Figure 6) [121].

### 4.2. Rheumatic Diseases

In the study of the pathogenesis of rheumatic diseases, the role of gasotransmitters is of considerable interest at present. The most studied common and socially significant diseases of the joints are osteoarthritis (OA) and rheumatoid arthritis (RA) [7].

**Figure 7:**
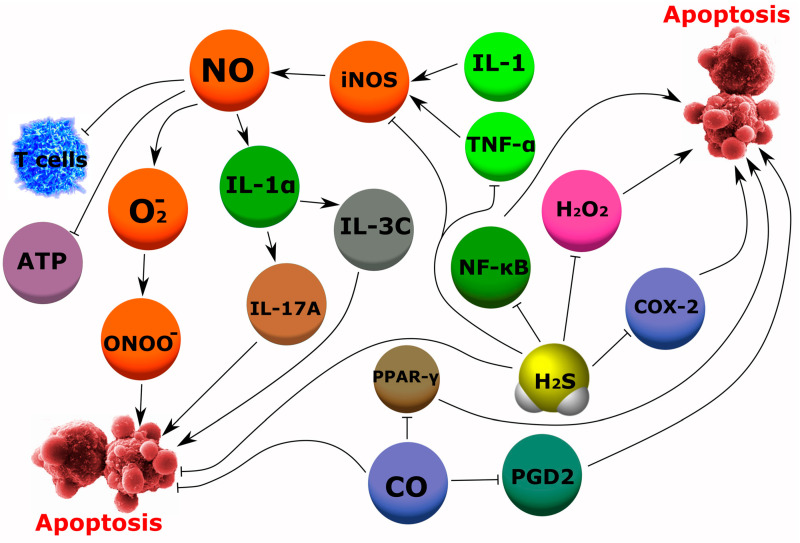
Possible gasotransmitter-dependent signaling mechanisms regulating apoptosis in diseases of the musculoskeletal system. NO, nitric oxide; CO, carbon monoxide; H_2_S, hydrogen sulfide; iNOS, inducible NO-synthase; ATP, adenosine triphosphate; T cells, lymphocytes; interleukins, IL-1α, IL-17A, and IL-3C; TNF-α, tumor necrosis factor alpha; O_2_^−^, superoxide anion radical; ONOO^−^, peroxynitrite; NF-κB, nuclear factor kappa-light-chain-enhancer of activated B cells; H_2_O_2_, hydrogen peroxide; COX-2, cyclooxygenase-2; PGD2, prostaglandin D2; PPAR-γ, peroxisome proliferator-activated receptor gamma. Arrows with a sharp end—positive regulation; arrows with a blunt end—negative regulation.

Thus, it has been shown that NO regulates T-cell function under physiological conditions, however, overproduction of NO can contribute to T-lymphocyte dysfunction. NO-dependent tissue damage has been associated with various rheumatic diseases, most commonly with rheumatoid arthritis [122]. In RA, the main source of NO are fibroblasts, osteoclasts, osteoblasts, endothelial cells, and immune cells such as macrophages and neutrophils. NO can cause dysregulation of the balance of osteoblasts and osteoclasts, and in combination with O_2_^−^ can be formed into ONOO^−^, which contributes to the degradation of articular cartilage and induces apoptosis. This leads to an imbalance in bone resorption and formation and damage to the joints [123]. Pro-inflammatory cytokines such as IL-1 and TNF induce the activation of iNOS in bone cells, resulting in overproduction of NO, causing bone loss (Figure 7). These actions of NO are relevant to the pathogenesis of osteoporosis in inflammatory joint diseases. Histomorphometric analysis of the bones of normal animals with bone loss caused by inflammation showed a profound depression of bone formation and signs of osteoblast apoptosis. These changes were not observed in iNOS knockout animals, suggesting that iNOS activation may contribute to the development of inflammatory osteoporosis as well as osteoblast apoptosis [124].

In addition to rheumatoid arthritis, NO is also involved in the pathogenesis of autoinflammatory joint diseases such as psoriatic arthritis (PsA) and systemic lupus erythematosus (SLE). In a mouse model of psoriasis and PsA induced by mannan, elevated levels of NO in the skin and extremities were found before the clinical onset of the disease. The generation of NO by local macrophages results in the release of IL-1α, which then activates IL-C3 to produce IL-17A, leading to increased disease severity (Figure 7) [125]. NOS expression was also increased in SLE patients [126]. Another disease in which NO may be involved in the pathogenesis is fibromyalgia, the etiology of which has not yet been fully established and is therefore of considerable interest. NO acts as a vascular smooth muscle relaxant, neurotransmitter, and immune regulator that sensitizes the spinal pain pathway. In fibromyalgia, there is an increase in ROS and a decrease in the antioxidant defense system. As a result, oxidative stress develops, which causes neuropathic pain and stimulates the development of chronic fatigue syndrome [127].

OA is a disease that has long been considered a primary metabolic disease, and mechanical cartilage degeneration has been the main element of pathogenesis. To date, the role of inflammation in the pathogenesis of OA is undoubted. NO also plays a role in osteoarthritis [128]. Analysis of the NO content in the synovial fluid of patients with OA has yielded conflicting results [129]. Unlike synoviocytes, chondrocytes have the ability to self-produce NO, as evidenced by increased levels of iNOS and NO in articular cartilage tissues. However, chondrocytes from patients without OA do not express iNOS, and experimental OA does not develop in iNOS knockout mice [130]. Some experiments have shown that NO by itself is not cytotoxic to cultured chondrocytes. However, excess NO can be detrimental, causing cartilage degradation or inhibiting cartilage matrix synthesis and causing mitochondrial dysfunction. There is a correlation between NO synthesis and the prevalence of apoptotic cells in cartilage in experimentally induced OA in rabbits. NO plays a role in mediating chondrocyte apoptosis, which is a common feature of progressive OA. Moreover, NO also alters the function of mitochondria in chondrocytes in OA, which leads to a decrease in cell survival by suppressing the activity of the mitochondrial respiratory chain and ATP synthesis [131]. The concentration of NO is significantly elevated in the synovial fluid in a model of OA in dogs [132] and humans [133]. However, there is evidence that NO has a beneficial effect on some cell types, including osteoblasts.

To a greater extent, H_2_S has a protective effect. It has been established that H_2_S has a cytoprotective effect through the modulation of antioxidant, anti-inflammatory, anti-apoptotic, and pro-angiogenic effects under various conditions [7]. The beneficial effect of H_2_S appears to be dose-dependent, as various studies have shown conflicting results [134]. In mouse macrophages, low concentration of H_2_S inhibited the activation and synthesis of several pro-inflammatory mediators such as TNF-α, NF-κB, IL-6, and IL-1β. However, at higher concentrations, H_2_S stimulated the production of pro-inflammatory molecules by human macrophages [135]. In addition, other studies have confirmed that H_2_S inhibits NF-κB-dependent expression of pro-inflammatory cytokines (e.g., IL-1β, IL-6, TNF-α) in macrophages, chondrocyte cell lines, and myoblast cell lines [136]. Interestingly, synovial fluid levels in RA were found to be higher than in patients with osteoarthritis, and the levels were positively correlated with disease activity and inflammation (Figure 7) [137]. The role of CSE in increased cartilage calcification has been revealed. Indeed, increased cartilage calcification is observed when CSE activity is suppressed, for example, in mouse models of age-related or surgically-induced OA. Calcification levels and histological severity of OA in mice and humans were negatively correlated with CSE expression. In vitro results have shown that CSE deficiency results in decreased cellular H_2_S levels and increased calcification in chondrocytes. With a pharmacological increase in the level of H_2_S in chondrocytes, a decrease in calcification was observed. These studies show that CSE generated is a regulator of experimental and human cartilage calcification [138,139,140]. H_2_S donors have shown significant anti-inflammatory effects in an osteoarthritis model and in rheumatoid arthritis in vitro and in vivo [141]. Increasing chondrocyte H_2_S production may represent a potential disease modifier for the treatment of OA. Over the past few years, it has become increasingly clear that H_2_S affects bone regeneration by acting on several levels, such as regulation of bone cell activity, reduction of oxidative stress, regulation of calcium consumption by bone cells, and promotion of angiogenesis. CBS and CSE are expressed in both multipotent stem cells and osteoblasts [142]. In particular, CSE is the predominant source of H_2_S in osteoblasts [143]. H_2_S plays a cytoprotective role in bone cells; it protects osteoblasts from homocysteine-induced mitochondrial toxicity [144] as well as from H_2_O_2_-induced apoptosis [145].

Only a few studies describe the molecular mechanism of H_2_S activity in healthy and diseased skeletal muscle. Bitar et al. [146] focused on evaluating the effect of H_2_S treatment on the development of sarcopenia. This loss of skeletal muscle mass and dysfunction has been described as a complication in diabetic patients, so studies were conducted using Goto Kakizaki rat models of diabetes with reduced systemic and muscle H_2_S bioavailability. The use of H_2_S donors increased muscle mass and reduced myostatistis levels. In animals with diabetes, the level of O_2_^−^ and H_2_O_2_ decreased [146]. In model organisms *C. elegans* with Duchenne muscular dystrophy, the level of H_2_S and the expression of genes necessary for sulfur metabolism are reduced. This decrease may be offset by an increase in the bioavailability of sulfur-containing amino acids, which increases lifespan, primarily by improving calcium regulation, mitochondrial structure, and slowing down muscle cell death [147].

CO mediates many of the biological effects that are attributed to HO, the enzyme responsible for CO production in mammals. The antioxidant and anti-inflammatory activity of HO-1 has been demonstrated in various disease models, including control of immune responses, production of inflammatory mediators, and mitigation of cartilage or bone destruction. Because HO-1 is highly expressed in the tissues of the joints of arthritic patients, it has been suggested that this pathway may play a protective role against degenerative joint diseases [148]. Low concentrations of CO are anti-inflammatory and may reduce bone erosion in an arthritis model. CO reduced RANKL expression in the synovium of arthritis mice. CO suppresses osteoclast differentiation by inhibiting RANKL-induced PPAR-γ activation (Figure 7). Considering the role of the PPAR-γ/cFos (AP-1) pathway in the regulation of the transcription factor NFATc1, a major regulator of osteoclastogenesis, further studies are needed to explore SO in the treatment of inflammatory bone diseases [149]. Experimental RA mice had elevated levels of anti-collagen antibodies, but decreased in the CO group. Histological analysis revealed a reduction in inflammation, erosion, and osteoclast counts only in CO-treated animals [150]. Ruthenium(II) tricarbonylchloro(glycinate) (CORM-3), releasing CO, reduced macroscopic signs of inflammation in the hind legs of OA mice, limited inflammatory cell migration and erosion of cartilage and bone, increased serum osteocalcin levels, and reduced PGD2 levels. In synovial tissues, a significant decrease in the expression of the genes of interleukin-1beta, receptor activator of nuclear factor kappa B ligand (RANKL), matrix metalloproteinase (MMP) 9, and MMP-13 were also revealed [151]. The study aimed to investigate the effect of carbon monoxide-releasing molecule 3 on osteoclastogenic differentiation of RAW264.7 cells and to investigate the possible mechanism underlying the regulatory effect. CORM-3 inhibits osteoclastogenic differentiation of RAW264.7 cells via CO release. The inhibitory effect is partially mediated by HO-1. The results suggest a potential application of CORM-3 in some bone defects [152].

### 4.3. Kidney Diseases

The prevalence of chronic kidney disease (CKD) in the population is increasing. Currently, the number of patients in the world suffering from CKD exceeds 850 million people [153]. The study of molecular mechanisms of kidney damage and the search for potential diagnostic markers as well as promising molecules with cytoprotective properties are of research interest. Of particular interest are such gasotransmitters as H_2_S, NO, and CO [154].

In recent decades, special attention has been paid to the study of the molecular role of H_2_S in kidney diseases. It has been established that CSE, CBS, and 3-3-MPST are localized in the glomeruli of the kidneys, tubular epithelium, and tubulointerstitium [155]. H_2_S regulates the excretory function, the release of renin from juxtaglomerular cells, thus controlling the activity of the renin–angiotensin–aldosterone system and blood pressure. H_2_S has a wide research potential. Despite the fact that its toxic properties were shown in earlier works [156], more and more scientific data have recently appeared on the study of its cytoprotective properties realized in various tissues, including the kidneys. The described effect is achieved due to antioxidant, anti-inflammatory, and anti-apoptotic actions [157,158].

**Figure 8:**
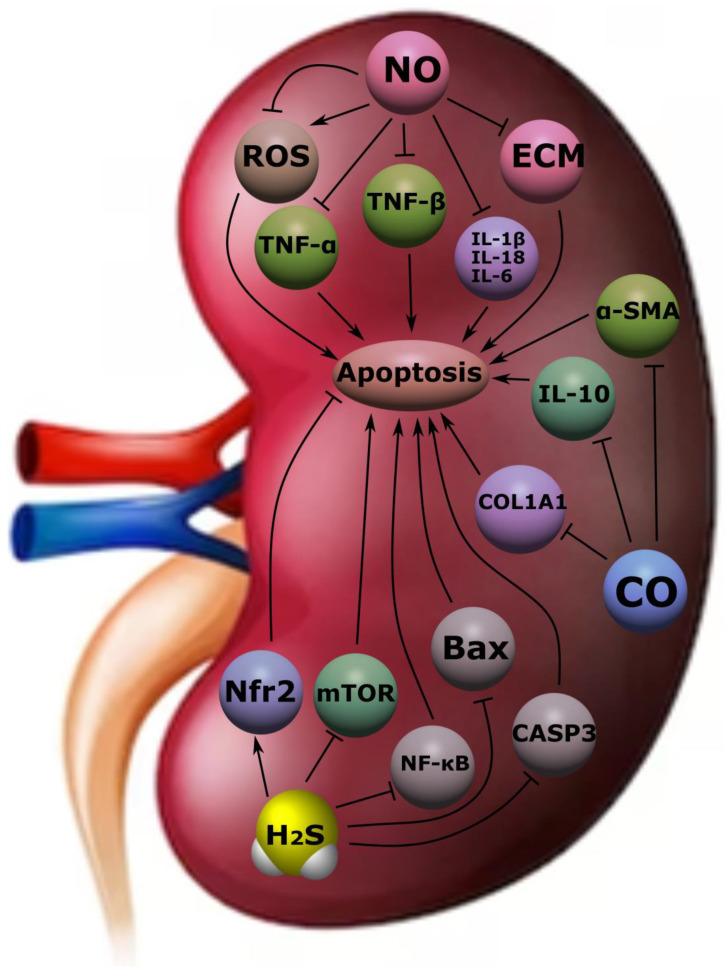
Possible gasotransmitter-dependent signaling mechanisms regulating apoptosis in kidney diseases. NO, nitric oxide; CO, carbon monoxide; H_2_S, hydrogen sulfide; ROS, reactive oxygen species; TNF-α and TNF-β, tumor necrosis factors; ECM, extracellular matrix; interleukins, IL-1β, IL-18, IL-10, and IL-6; α-SMA, smooth muscle alpha-actin; COL1A1, type 1 collagen; Bax, bcl-2-like protein 4; Nfr2, nuclear factor erythroid 2-related factor 2; mTOR, mammalian target of rapamycin; CASP3, cysteine-aspartic acid protease 3; NF-κB, nuclear factor kappa-light-chain-enhancer of activated B cells. Arrows with a sharp end—positive regulation; arrows with a blunt end—negative regulation.

Acute kidney injury (AKI) is a rapidly progressive renal dysfunction characterized by a rapid increase in creatinine and decreased urine output lasting from hours to days [159]. The causes of AKI can be various factors, including sepsis, glomerulonephritis, medication (e.g., NSAIDs, cisplatin), liver failure, heart failure, ischemia and reperfusion syndrome, etc. [160]. The latter factor is one of the most frequent in the development of this pathological condition and is associated with the development of fibrosis and inflammation in the kidneys and, as a result, acute impairment of their function [161]. H_2_S plays an important role in this process and performs various functions depending on the rate of its formation. So, at a high level, it induces the synthesis of pro-inflammatory mediators (IL-1β, IL-6, TNF-α, prostaglandin E2, and NO), whereas at low concentrations it exhibits cytoprotective properties and inhibits their formation, acting as an antioxidant agent [162]. The anti-inflammatory effect is also supported by the suppression of H_2_S activity of NF-κB [163]. At the same time, endogenous H_2_S realizes its anti-inflammatory and anti-apoptotic potential through inhibition of Toll-like receptors in the renal tubular epithelium [164]. In an experiment on rats with lipopolysaccharide-induced AKI/sepsis-associated AKI, it was demonstrated that H_2_S prevented the development of inflammation and oxidative stress by reducing the expression of TNF-α, IL-1β, MDA, MPO, H_2_O_2_, and caspase-1, as well as through inhibition of the TLR4/NLRP3 signaling pathway (Figure 8) [165].

In patients with chronic kidney disease (CKD), a decrease in H_2_S levels was found. In experimental work on nephrectomized rats, NaHS exerted antioxidant, antiapoptotic, and anti-inflammatory effects through Nfr2 activation and downregulation of the mammalian target of rapamycin (mTOR), which generally had a positive effect on kidney function [166]. At the same time, NaHS realizes these effects through MAPK and NF-κB, suppressing inflammation and apoptosis. In mice with adenine-induced CKD, NaHS suppressed the production of TNF-α, IL-6, IL-10, NF-κB, MCP-1, MDA/SOD, GSH-Px, p-MAPK, Bax, cleaved caspase-3, and Bcl-2 (Figure 8) [167]. Low levels of H_2_S negatively affect kidney function and contribute to the acceleration of the progression of CKD due to increased autophagy, apoptosis, development of oxidative stress, and inflammation. An increase in H_2_S levels may have a nephroprotective effect and slow down the discussed processes [168].

The progression of CKD is associated with the development of fibrotic processes in the kidneys. This is observed in diabetes mellitus, arterial hypertension, glomerulonephritis, and other diseases [165]. The accumulation of extracellular matrix leads (ECM) to impaired renal function. It has been demonstrated that administration of H_2_S to mice with streptozotocin (SZT)-induced obesity reduced the accumulation of type II collagen, tissue inhibitor of metalloproteinase 2, and hydroxyproline in the kidneys and suppressed the activity of connexins and MMP 1/2 [169]. Administration of NaHS to diabetic mice reduced serum levels of creatinine, urea nitrogen, and pro-inflammatory cytokines and inhibited the activation of the TGF-β1/Smad 3 pathway. The anti-inflammatory effects of H_2_S, described above, slow the rate of renal fibrosis and CKD progression [170].

H_2_S also plays an integrative role in other pathological conditions. Thus, in obstructive nephropathy, there is a decrease in the expression of CSE, CBS, and 3-MPST, which increases the risk of tubulointerstitial fibrosis [171,172]. In mice with induced hyperhomocysteinemia, there is a decrease in CSE and CBS levels, whereas H_2_S reduces the concentration of homocysteine [173]. The latter in turn induces kidney damage. Hydrogen sulphide also has a nephroprotective effect when using nephrotoxic drugs such as cisplatin, paracetamol, gentamicin, etc. [174,175,176]. The main defense mechanisms are the suppression of inflammation, apoptosis, and oxidative stress.

NO, which is considered one of the most studied gasotransmitters, plays a key role in various physiological and pathological processes and implements its effects in the kidneys as well [8]. With glomerulonephritis, immune inflammation develops and damage to the structures of the glomeruli of the kidneys, in particular, mesangial cells and podocytes, occurs. In experimental work on rats during the cultivation of mesangial cells, the introduction of NO donators (spermine, NOC-18, and SNAP) suppressed the expression of profibrogenic genes at the transcriptional level, which confirmed the antifibrotic effect of NO [177]. In another study, it was shown that the use of L-arginine in animals with ATS-glomerulonephritis slows down the process of kidney fibrosis induced by the suppression of TGF-β. At the same time, one of the causes of kidney fibrosis is the excessive formation of ECM. In mesangial cells, NO regulates the production of its components (MMP-9, MMP-13, PAI-1, TIMP-1), reducing renal failure [178,179].

At different concentrations, NO, like H_2_S, can exhibit different effects. Thus, an increase in NO levels can inhibit mitochondrial respiration. On the other hand, AKI is characterized by NO deficiency, which contributes to the progression of kidney damage and supports the transformation of AKI into CKD and the development of arterial hypertension [180]. It was found that the NO-donator EDV regulates oxidative stress and lipid peroxidation in the kidney tissue and the inflammatory process by suppressing the activity of IL-1β, IL-18, IL-6, and TNF-α (Figure 8) [181].

CO is one of the first gasotransmitters. It is involved in a number of physiological and pathological processes. Thus, in AKI caused by obstructive causes, the use of CO in mice reduced the phenomena of fibrosis and prevented kidney damage. This was associated with a decrease in ECM and downregulation of α-SMA, type I collagen, and fibronectin expression in the kidney [182]. At the same time, the MKK 3 signaling pathway is the main one at the stage of implementation of these effects. CO has several important functions that are indirectly related to the functioning of the kidneys. Among them: participation in angiogenesis, the development of vasodilation, a decrease in platelet aggregation, the induction of an inflammatory process, etc. [167,183]. Despite the known toxic effect of CO at high levels, its low concentrations may have a cytoprotective effect. CO has an anti-inflammatory effect by blocking TNF activity and potentiating the expression of the anti-inflammatory cytokine IL-10 (Figure 8) [184,185]. The anti-inflammatory and anti-apoptotic properties of CO are also used in transplantology. CO suppresses oxidative stress, mRNA expression of pro-inflammatory cytokines, inhibits apoptosis of the epithelium of the tubules of the kidney graft, and suppresses interstitial fibrosis. These effects are achieved by the increased expression of phosphatidylinositol-3 kinase and phosphorylation of Akt and mitogen-activated protein kinase p38 [156].

### 4.4. Neurodegenerative Diseases

Neurodegenerative diseases (ND) are a serious problem in the global health system. They cause severe disability and death for millions of people around the world. The most striking examples of neurodegenerative diseases (ND) are Alzheimer’s disease (AD), Parkinson’s disease (PD), and amyotrophic lateral sclerosis (ALS) [186].

**Figure 9:**
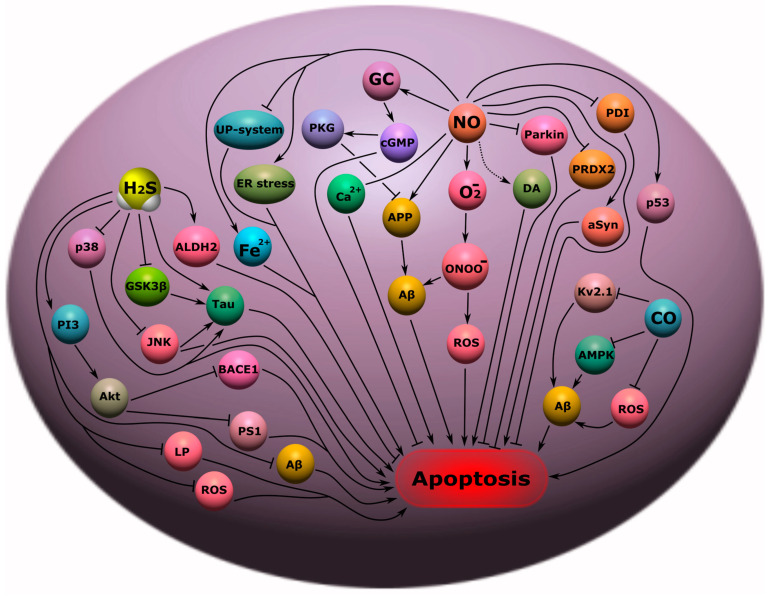
Possible gasotransmitter-dependent signaling mechanisms regulating apoptosis in neurodegenerative diseases. NO, nitric oxide; CO, carbon monoxide; H_2_S, hydrogen sulfide; ROS, reactive oxygen species; O_2_^−^, superoxide anion radical; ONOO^−^, peroxynitrite; GC, guanylyl cyclase; cGMP, cyclic guanosine monophosphate; PKG, protein kinase G; Ca^2+^, calcium ions; APP, amyloid-beta precursor protein; Aβ, amyloid beta; UP-system, ubiquitin-proteasome system; ER stress, endoplasmic reticulum stress; Fe^2+^, iron ion; p38, p38 mitogen-activated protein kinases; GSK3β, glycogen synthase kinase-3 beta; ALDH2, aldehyde dehydrogenase 2; PI3, phosphorus triiodide; Akt, protein kinase B; LP, lipid peroxidation; JNK, c-Jun N-terminal kinases; Tau, microtubule-associated protein tau; BACE1, beta-site APP cleaving enzyme-1; PS1, presenilin-1; Parkin, E3 ubiquitin ligase; DA, dopamine; PDI, protein disulfide-isomerase; PRDX2, peroxiredoxin 2; aSyn, alpha-synuclein; Kv2.1, voltage-gated potassium channel KV 2.1; AMPK, AMP activated protein kinase. Arrows with a sharp end—positive regulation; arrows with a blunt end—negative regulation.

Potential molecular targets in NS can be gasotransmitters. Thus, it was shown that NO can be a key molecular player in the pathogenesis of AD, characterized by the loss of synapses and neurons, and as a result, memory impairment, cognitive decline, and a tragic ending—death. Most often, NO was associated with neurotoxic damage in AD, however, as it turned out later, its role is far from being so unambiguous in this pathology. Of course, high concentrations of NO lead to nitrosyl stress, with the formation of an extremely aggressive peroxynitrite radical (ONOO^−^), which has a pronounced cytotoxic effect. The pathway of NO/O_2_^−^/ONOO^−^-induced apoptosis of neurons has been demonstrated in various experimental models of AD (Figure 9) [187]. However, it should be noted that such a neurotoxic effect of NO most often develops when iNOS is overexpressed, which generates high concentrations of NO. Constitutive forms of NOS, on the contrary, can have cytoprotective effects, in particular, due to the induction of the cGMP pathway, which causes an increase in cerebral blood supply, a decrease in oxidative stress, and Ca^2+^ excitotoxicity in AD (Figure 9) [1].

Of great interest is the NO-dependent regulation of the level of the key AD protein, β-amyloid precursor protein (APP), which is responsible for the formation of amyloid plaques and neuronal death. NO can modulate the level of APP through the amyloidogenic pathway of processing depending on its concentration, leading to either its activation or inhibition. The NO-mediated anti-amyloidogenic effect was due to signaling through GC/cGMP/PKG, and the amyloidogenic activity of NO at high concentrations was mediated through mechanisms associated with ONOO^−^ [188]. In addition, nitrosyl stress has been shown to lead to β-amyloid (Aβ)-induced neurotoxicity, which underlies the pathogenesis of AD (Figure 9) [189].

NO is involved in the pathophysiological processes associated with PD. High levels of nNOS and iNOS expression were found in the substantia nigra (SN) of patients and animals with PD [2]. It is indicated that nitrosyl stress is one of the main causes of degeneration of dopaminergic neurons in PD. In addition, NO can lead to abnormal dopamine metabolism with the formation of toxic metabolites leading to nerve cell death [190]. Stress of the endoplasmic reticulum and disruption of the ubiquitin-proteasome system is also one of the effects of NO in this pathology [191]. Overproduction of NO leads to neuronal damage by S-nitrosylation or nitration of several important proteins, including S-nitrosylation of parkin, protein disulfide isomerase, mitochondrial complex I, peroxiredoxin-2, and nitration of α-synuclein in PD (Figure 9) [192]. Additionally, NO disrupts iron homeostasis in neurons, causing its accumulation through a decrease in APP expression in PD models, which leads to the death of dopaminergic neurons [193].

There is an increase in NO and its metabolites in the cerebrospinal fluid of patients with ALS. NO plays a key role in glutamate-induced neuronal death in ALS [194]. Degenerative neurons of the anterior horns of the spinal cord in ALS showed a high expression of nNOS, which may be associated with their subsequent death [195].

H_2_S is also involved in AD. H_2_S has been shown to bind to Tau proteins, the main components of neurofibrillary glomeruli, and enhances their catalytic activity. H_2_S prevents Tau hyperphosphorylation by sulfhydration of GSK3β (Figure 9). Administration of H_2_S donors to AD mice improved motor and cognitive impairments in AD [196]. It should be noted that the level of H_2_S was reduced in patients with AD compared with normal, and there was a correlation of a decrease in the concentration of H_2_S with the progression of the disease [197]. In addition, H_2_S induced the expression of aldehyde dehydrogenase 2 and reduced the formation of lipid peroxidation products in the hippocampus of AD rats [198]. Additionally, H_2_S donors reduce the activity of JNK and p38 (Figure 9), which play a key role not only in the phosphorylation of Tau, but also in inflammation and apoptosis [199]. This messenger reduces the level of homocysteine, a high level of which increases the risk of developing AD, and is a negative concomitant factor of this pathology [200]. The H_2_S donor decreased BACE1 and PS1 levels via the PI3/Akt pathway and also decreased Aβ in APP/PS1 transgenic mice (Figure 9) [201].

An equally important role of H_2_S is in PD. Studies have shown that parkin sulfhydration decreases in PD (Figure 9), leading to a decrease in its catalytic activity [202]. In a mouse model of PD, H_2_S demonstrated a reduction in the loss of dopaminergic neurons and promoted adult neurogenesis by regulating the Akt/GSK-3β/β-catenin cascade (Figure 9) [203]. On a cell culture treated with 1-Methyl-4-phenylpyridinium ion (MPP+) used to model PD, it was shown that the use of an H_2_S donor led to a decrease in the expression of pro-apoptotic proteins caspase 3, Bax, and products of lipid peroxidation and the inhibition of the NO-ROS pathway (Figure 9) [4]. In a 6-hydroxydopamine (6-OHDA)-induced PD rat model, administration of an H_2_S donor resulted in the inhibition of microglial activation in the SN, accumulation of pro-inflammatory factors, and a decrease in malondialdehyde [204]. In addition, inhaled H_2_S in 1-methyl-4-phenyl-1,2,3,6-tetrahydropyridine (MPTP)-induced PD mice prevented neuronal apoptosis and nigrostriatal gliosis [205].

ALS correlated with high levels of H_2_S in the cerebrospinal fluid of patients suffering from this disease. It is known that a high concentration of H_2_S can lead to cytotoxic effects. It is assumed that in ALS H_2_S is responsible for the death of neurons through the activation of the mechanisms of Ca^2+^ excitotoxicity. Thus, the addition of H_2_S to a spinal culture obtained from mice with ALS led to Ca^2+^ overload of cells and their death [206]. However, H_2_S can activate the mechanisms of antioxidant and anti-inflammatory protection in ALS [82].

It is reported that CO can protect neurons from apoptosis in AD. HO-1 is known to be highly expressed in patients with Alzheimer’s disease, exerting a neuroprotective effect. HO-1/CO has been shown to protect cells from the toxicity of protofibrillar Aβ_1-42_ by inhibiting AMPK activation and possibly also by reducing K^+^ efflux through Kv 2.1 K^+^ channels (Figure 9) [207]. CO can interfere with Aβ_1-42_-dependent astrocyte death by reducing oxidative stress levels [208].

It is worth noting the role of these gasotransmitters in one of the key mechanisms of neurodegeneration—in the processes associated with axon degradation, followed by its rupture, i.e., axotomy. Axotomy, i.e., the complete cutting of an axon, is one of the classic models of neurodegeneration [209]. In our studies, we were able to show that the NO-donor sodium nitroprusside causes pronounced nuclear deposition of p53 in neurons and glial cells and their apoptotic death during axotomy in vertebrates. Use of a selective iNOS inhibitor S-methylisothiourea hemisulfate led to the opposite effect. Using a simple model of axotomy—the crayfish stretch receptor, which consists of two mechanoreceptor neurons surrounded by a sheath of satellite glia and a pair of receptor muscles—we examined in detail the intracellular processes associated with the generation of NO in photosensitized cells [10]. These studies have shown the key role of NO in the regulation of survival and death of neurons and glial cells in neurotrauma.

## 5. Molecular Mechanisms of Gasotransmitter-Dependent Apoptosis in Neuropsychiatric Diseases

### 5.1. Schizophrenia

**Figure 10:**
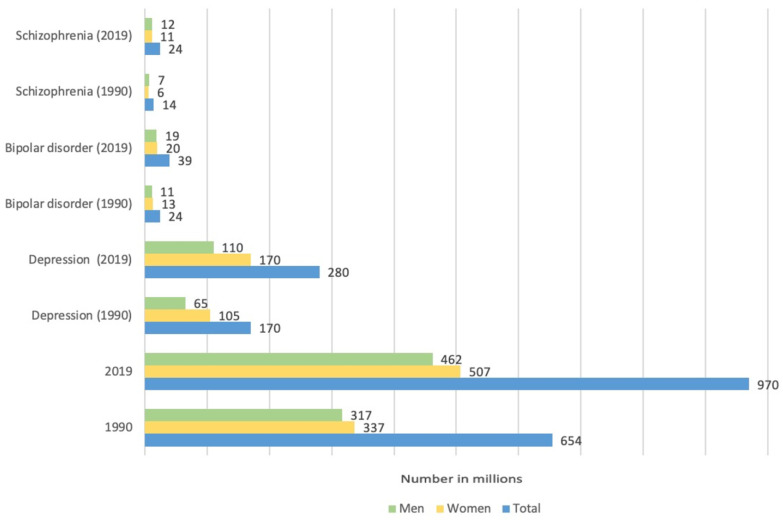
The incidence of mental disorders in the world from 1990 to 2019.

Schizophrenia (SCZ) affects approximately 24 million people, or 1 in 300 people worldwide (Figure 10). SCZ is characterized by a variety of psychopathological symptoms: productive (delusions, hallucinations, psychomotor agitation) and negative symptoms (loss of motivation, desire and volitional drive) as well as cognitive impairment [210,211,212,213,214].

For a long time, this pathology was considered from the perspective of impaired development of the nervous system. However, the accumulated experimental experience of subtle neurostructural changes after the onset of psychosis has led to the suggestion that apoptosis may be one of the mechanisms of SCZ pathogenesis [215,216].

It is known that activation of apoptosis can cause rapid and total death of neurons and glial cells in the brain. In addition, proapoptotic signaling can cause non-lethal changes in neurons, characterized by neurite degeneration and synapse elimination. For example, caspase-dependent processes can be realized not only in the cell death program, but also manifest themselves in local changes in cell architectonics [217,218]. To date, a number of pathomorphological processes have been identified in SCZ, manifested in cortical atrophy [219], decreased myelination, glial abnormalities [220], reduced synaptic density, and degradation of dendrites and axons [221]. All these negative changes are due to biochemical and molecular genetic changes in SCZ, including dysregulation of signaling pathways that control apoptosis [215,216,220]. Unfortunately, so far there is no single concept in this area; humanity has only slightly revealed the seeding of the secrets of the molecular mechanisms underlying the pathogenesis of this disease.

**Figure 11:**
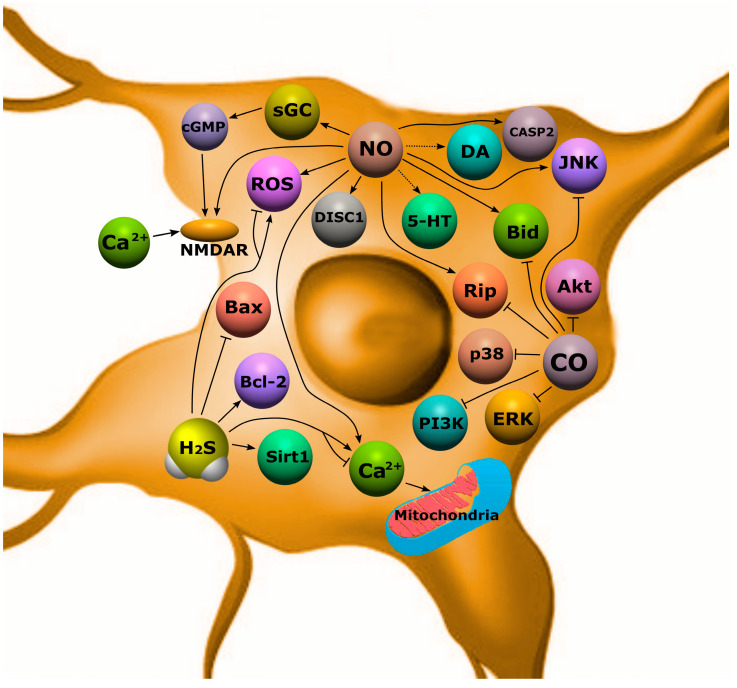
Possible gasotransmitter-dependent signaling mechanisms regulating apoptosis in mental disorders. NO, nitric oxide; CO, carbon monoxide; H_2_S, hydrogen sulfide; ROS, reactive oxygen species; CASP2, cysteine-aspartic acid protease 2; Bax, bcl-2-like protein 4; DA, dopamine; 5-HT, serotonin; sGC, soluble guanylyl cyclase; cGMP, cyclic guanosine monophosphate; p38, mitogen-activated protein kinase p38; JNK, c-Jun N-terminal kinases; Bid, BH3 interacting-domain death agonist; Rip, ribosome-inactivating protein; Akt, protein kinase B; ERK, extracellular signal-regulated kinase; PI3K, phosphoinositide 3-kinases; Sirt1, NAD-dependent deacetylase sirtuin-1; Bcl-2, B-cell lymphoma 2; NMDAR, N-methyl-D-aspartate receptor; DISC1, disrupted in schizophrenia 1. Arrows with a sharp end—positive regulation; arrows with a blunt end—negative regulation; dashed arrows—modification/adjustment.

At present, it is known that gasotransmitters are involved in the pathogenesis of SCZ. So, for the first time, in the 1970s, Averbukh et al. as well as Bulba et al. suggested that there is a relationship between NO and SCZ. However, this topic has been studied most intensively in the last two decades. Post-mortem studies have shown an increase in NO and nNOS in the brain of people with SCZ [5]. Other studies report that the levels of Ca^2+^-dependent NOS did not differ from the control group, but their activity was significantly reduced [222]. In addition, there are data indicating a deficiency of NO production in patients with SCZ [223]. The role of NO in this pathology is often considered as an inducer of oxidative stress, which is an important negative mechanism in SCZ. In patients with SCZ, there is a violation of the antioxidant defense system, regulation of redox transcription factors, and an increase in lipid peroxidation products [224]. It is assumed that NO hyperproduction can enhance these processes, acting as a negative regulator of the survival of neurons and oligodendrocytes in SCZ [5]. However, recent research has shown that the NO donor, sodium nitroprusside, reduced psychotic symptoms in animal models of SCZ, possibly through the activation of the NMDA-nNOS-cGMP pathway with improved cerebral blood flow [223]. In addition, NO can activate NMDA receptors, the activity of which is significantly reduced in SCZ, which leads to a mismatch of intra- and intercellular signaling communications (Figure 11) [225].

Disrupted-in-Schizophrenia 1 (DISC1) is one of the key proteins associated with the development of schizophrenia and other psychiatric disorders. It is a conserved 93.6 kDa protein with globular N-terminal, helical C-terminal, and several helical–helical domains. These architectonics allow DISC1 to interact with various proteins, including signal proteins responsible for various biological effects, incl. cell death (Figure 11) [226]. It has been shown that NO can influence the interaction of DISC1 with NDEL1 by controlling the growth of neurites in the prefrontal cortex [227]. Schizophrenia is characterized by impaired functioning of various neurotransmitters, for example, dopamine (DA), glutamate, acetylcholine, serotonin, and GABA, which leads to disorganization of the functioning of neural networks, neuroglial interaction, and as a result, the death of neurons and glial cells. It is known that NO and its active metabolites can modify various molecular targets, including neurotransmitters [223,228]. For example, the chemical interaction of NO with dopamine is a source of neurotoxins that promote neurodegeneration [190]. NO can interact with serotonin to form 4-nitroso-serotonin and 4-nitro-serotonin, which do not have a neuromodulatory effect. Changes in the serotonin–dopamine balance in the brain is one of the key mechanisms in the pathogenesis of SD. In addition, serotonin can modulate dopamine levels, as well as reduce free radical-induced neuronal death [228].

NO-induced apoptosis of neurons and glial cells in schizophrenia may be due to the development of mitochondrial dysfunction, which leads to the release of proapoptotic factors from mitochondria into the cytoplasm [229]. It is noted that in schizophrenia the level of the proapoptotic protein Bax increases and the expression of the antiapoptotic protein Bcl-2 decreases in the temporal cortex. An increase in the expression of proapoptotic proteins JNK, caspase-2, Rip, and Bid in the hippocampus was also observed [230]. NO can modulate the expression of these proteins by both direct binding to them at specific sites and through activation or inhibition of various signaling pathways [34,36].

Equally interesting, but not as numerous, are studies on the role of H_2_S in SCZ. It is believed that excess production of H_2_S may underlie the pathogenesis of schizophrenia. It was shown that in post-mortem brain samples of patients with SCZ, the level of MPST and CBS was increased due to the coordinated activation of several genes that regulate the processes associated with the formation of H_2_S_._ The level of MPST positively correlates with the severity of symptoms in SCZ. Activation of MPST and CBS and their concomitant accumulation of sulfides in the brain can induce schizophrenic behavior. Under conditions of sulfide stress, the psychotic symptoms characteristic of SCZ may be exacerbated. It was also demonstrated that in neuronal cells obtained from patients with SCZ, CBS mRNA was significantly increased relative to control samples. This indicates that systemic H_2_S production may be elevated early in CNS development in SCZ. Excessive synthesis of H_2_S reduces the expression of energy metabolism genes, disrupting the mitochondrial processes of bioenergetics. In addition, C3H mice, which exhibit a greater propensity for schizophrenic behavior, were found to have elevated MPST levels and greater sulfide deposition relative to controls [231].

However, the role of H_2_S in this pathological condition is not so clear. Studies have shown that a decrease in the level of H_2_S in blood plasma in patients with SCZ correlated with psychopathological and cognitive impairments [232]. Along with this, H_2_S can affect the release of glucose, lactate, and glutamate in the hippocampus, acting as an activator of the metabolism of neurons and glial cells in SCZ. It is also known that in SCZ there are significant changes in the activity of the hypothalamic–pituitary–adrenal system in relation to the secretion of cortisol. The use of CBS and CSE inhibitors led to a decrease in corticosterone secretion in response to adrenocorticotropic hormone of the adrenal gland, which may be associated with an intensification of mitochondrial oxidative stress [233].

### 5.2. Depression

In 2019, 280 million people were affected by depression, including 23 million children and adolescents (Figure 10). Depression is characterized by a decrease in mood or a loss of interest in any activity [234,235].

For a long time, depression was studied by traditional descriptive methods of psychiatry. However, recent advances in modern science have made the neuroimaging of the subtle molecular mechanisms that occur in this disorder possible. Evidence was obtained that various pathomorphological changes occur in the brain in depression, associated with a violation of biochemical and molecular genetic processes in neurons and glial cells. Depression may be based on such pathological processes as oxidative stress, damage to proteins, lipids, nucleic acids, as well as cell death, including apoptosis [236,237]. Recently, more and more information has appeared on the role of various gasotransmitters in the pathogenesis of depression.

It is known that NO is involved in various pathological processes, including mental disorders. This messenger has been proposed as an important signaling agent in the pathogenesis of depression. The first evidence for this hypothesis came in 1980, when the NOS inhibitor methylene blue was shown to have an antidepressant effect. Ten years later, it was proved that NO plays one of the key roles in the pathogenesis of depression [238]. This concept is based on studies that have documented the activity of nNOS and eNOS in the prefrontal cortex [222], the total number of NOS-immunoreactive paraventricular neurons [3], as well as the level of NO and its metabolites in plasma [239,240] in patients with depression. However, there are enough studies reporting high levels of NO and its metabolites in depression [241,242,243].

nNOS is known to be localized in areas of the brain associated with stress responses and depression. The limbic–hypothalamic–pituitary–adrenal (LHPA) is one major system involved in depression. NO can regulate LHPA [244]. Thus, NO can modulate norepinephrine, serotonin, and dopamine [245]. Disruption of the levels of these neurotransmitters can lead to cell death [246].

High levels of NO can cause oxidative stress, which is the main cause of neurodegenerative changes in the pathogenesis of most depression [247]. Studies indicate that against the background of elevated NO levels, a significant weakening of the enzymatic links of the antioxidant defense system occurs in patients suffering from depression [248]. It was also found that the level of Bax in the olfactory bulb (OL) increased against the background of a decrease in the expression of the Bcl-2 protein in rats with depression (Figure 11). Olfactory impairment and decreased OL volume are often observed in patients with depression [249]. Studies report that NO can control neurogenesis and cell death in this part of the brain [250]. An increase in proapoptotic factors is characteristic of depression [251]. However, the role of NO is still unclear in the development of cell death in depression.

In turn, H_2_S is also an important molecular player in depression. H_2_S has been found to have antidepressant and anxiolytic effects. One of the mechanisms of these effects may be H_2_S-dependent modulation of the level of activity of NMDA receptors, the functioning of which is impaired in depression. Treatment with H_2_S donors increased synaptic plasticity and cognitive function. A decrease in the level of H_2_S in blood plasma correlated with the severity of depression [252]. In addition, H_2_S can regulate the expression of Sirt1, an enzyme that deacetylates transcription factors and one of the main proteins of mood disorders. Sirt1 has anti-apoptotic activity through deacetylation of p53. Inhibition of Sirt1 reduced the protective effects of H_2_S against sleep deprivation-induced depressive and anxious behavior [253]. An increase in the expression of p-P38, p-ERK 1/2, PI3K, and p-Akt in the prefrontal cortex of mice with depression caused by chronic pain was also noted. The use of an H_2_S donor led to a decrease in the level of these proteins (Figure 11) [254]. H_2_S has also been reported to inhibit inflammation and ferroptosis in depression [255].

The role of another gasotransmitter, CO, has also been found in the pathogenesis of depression. The use of the HO-1 activator resulted in a decrease in the level of p-P38, p-ERK 1/2, PI3K, and p-Akt in a mouse model of depression [254]. It has also been reported that CO may reduce negative symptoms in depression. The decrease in these symptoms may be due to CO effects implemented in the corticolimbic system. So, it is known that CO/HO-1 can affect the level of trophic factors in the amygdala, hippocampus, and also in the frontal cortex, which can help improve the emotional state [256]. It has been shown that CO can induce dopamine release from presynaptic terminals [257].

### 5.3. Bipolar Disorder

Bipolar disorder (BD) is a chronic mental illness characterized by a phasic change in mood: a “swing” between mania and depression. In 2019, 40 million people suffered from bipolar affective disorder (BAD). People with bipolar disorder have alternating depressive and manic symptoms [258,259].

Despite global studies conducted over the past decades, the molecular–cellular mechanisms underlying this pathology still remain unclear. One of the mechanisms of BD pathogenesis may be a decrease in the density of neurons and glial cells in the frontal and subcortical areas of the brain as a result of increased apoptosis and oxidative stress. There is also a pronounced tendency for a decrease in the morphometric characteristics of these cells in BD [260]. Using electron microscopy, signs of apoptosis of oligodendrocytes in BD have been detected [261]. A number of studies have reported activation of proapoptotic signaling in neurons characterized by an increase in the expression of Bax, caspase-9, and caspase-3 [262] and a decrease in the levels of Bcl-2 and BDNF (Figure 11) [263].

A number of studies point to the role of NO in the pathogenesis of BD. A high level of NO against the background of a violation of the antioxidant defense system is characteristic of this disease [264]. However, studies have shown that the number of neurons expressing nNOS is markedly reduced in BR in the locus coeruleus containing a large population of noradrenergic neurons. In turn, disruption of the norepinephrine system is involved in the pathophysiology of affective disorders. It is known that a disturbance in the synthesis of NO can lead to a decrease in the synthesis of noradrenaline [265]. In addition, NO can modulate the level of proapoptotic proteins in BD. There is also a violation of the H_2_S-synthesizing system in BD [252].

### 5.4. Anxiety Disorders

Anxiety disorders are a group of disorders characterized by irrational, uncontrollable fear and persistent feelings of anxiety. This group of anxiety disorders includes obsessive–compulsive disorder, panic disorder, generalized anxiety disorder, post-traumatic stress disorder (PTSD), etc. [266].

To date, it is known that an increase in apoptosis in the nervous tissue can be observed in anxiety states [267,268]. Thus, in particular in PTSD, an increase in apoptosis was observed in the hippocampus, amygdala, and other areas of the brain. It is indicated that PTSD enhances neuronal apoptosis due to the activation of caspases, disruption of myelination processes, and axon growth [267,269]. In the PTSD model in rats, an increase in the expression of caspase-9, caspase-3, and cytochrome c and a decrease in the Bcl-2/Bax ratio were observed (Figure 11). Using the TUNEL method, it was shown that PTSD induces neuronal apoptosis [268]. Gasotransmitters, including NO, CO, and H_2_S, may be involved in intracellular processes in anxiety disorders [270,271].

NO can induce neuronal apoptosis during stress-induced anxiety through increased levels of c-Fos, microglial activation, and oxidative stress [272]. It is believed that an increase in the level of c-Fos is observed during stress reactions in various areas of the brain responsible for fear and anxiety [273]. This protein causes prolonged excitation of neurons, which can lead them to apoptosis [274]. In addition, mental stress is accompanied by an increased release of corticosterone, norepinephrine, and adrenaline, which lead to massive activation of microglia and cell death [275]. NO has been shown to regulate catecholamine levels [276]. At the same time, activated microglia itself is a source of NO [277]. Inhibition of iNOS in a rat model of anxiety reduced inflammation and oxidative stress [278].

CO has been reported to exert an anxiolytic effect in rats via a cGMP-dependent heme oxygenase pathway at the locus coeruleus (LC), which is one of the most important noradrenergic centers in the brain [279]. It is worth noting that a high level of HO-2/HO-1 is observed in LC neurons. This points to an important CO-dependent mechanism in the regulation of LC nerve cells [280].

H_2_S also plays an important role in anxiety disorders. H_2_S donors have been shown to reduce oxidative stress in anxiety-induced rats [281,282]. In addition, H_2_S can maintain the level of glutathione in the nervous system, facilitate neuroplasticity in the amygdala, and regulate the level of intracellular Ca^2+^ in neurons and glial cells in anxiety states [283].

## 6. Conclusions

Gasotransmitters have a wide range of biological effects in normal and pathological conditions, including internal diseases and mental disorders. Their role is rather controversial in these diseases, and the molecular mechanisms of the regulation of apoptotic signaling are not well understood. However, gasotransmitters are already promising molecular targets for the treatment and prevention of the discussed diseases. The molecular mechanisms of the interaction of these messengers and feedbacks are of research interest and still hold many hidden possibilities for studying the regulation of signaling pathways associated with them.

Currently available scientific data point to the direct role of gasotransmitters in the regulation of expression and localization of anti- and pro-apoptotic proteins, ion channel activity, intracellular homeostasis, energy metabolism, lipid peroxidation, and antioxidant protection in internal diseases and mental disorders. Further study of gasotransmitter-dependent signaling mechanisms will allow a better understanding of the fundamental mechanisms of cell survival and death under conditions of stress reactions and may also form the basis for the development of clinically effective cytoprotective drugs of a new generation.

## Data Availability

Not applicable.
